# Lenalidomide at Therapeutic and Supratherapeutic Doses Does Not Prolong QTc Intervals in the Thorough QTc Study Conducted in Healthy Men

**DOI:** 10.1111/bcpt.12081

**Published:** 2013-06-20

**Authors:** Nianhang Chen, Ying Ye, Liangang Liu, Josephine Reyes, Mahmoud S Assaf, Claudia Kasserra, Simon Zhou, Maria Palmisano

**Affiliations:** Department of Clinical Pharmacology, Celgene CorporationSummit, NJ, USA

## Abstract

The effect of lenalidomide on the corrected QT (QTc) interval was evaluated in healthy men and extended to patients based on the lenalidomide concentration–QTc (C–QTc) relationship. A rigorous assessment of the effect of lenalidomide on QTc intervals was conducted in healthy volunteers who each received, in randomized order, a single oral dose of 10 mg lenalidomide, 50 mg lenalidomide, 400 mg moxifloxacin (positive control) and placebo. Plasma lenalidomide exposure was compared between healthy volunteers and patients with multiple myeloma or myelodysplastic syndromes. In healthy volunteers, moxifloxacin produced the expected significant prolongation in QTcI (individual correction). For lenalidomide 10 mg and 50 mg, the time-matched changes from placebo in the baseline-adjusted least-squares mean QTcI were <3 ms with the upper limit of the two-sided 90% confidence interval for the change <10 ms at all time-points. No subjects experienced QTcI >450 ms or change from baseline >60 ms after lenalidomide administration. Similar results were seen with QT interval data corrected by Fridericia and Bazett methods. The C–QTc analysis yielded no significant association between lenalidomide concentrations and QTcI changes up to 1522 ng/mL; this range was close to that observed in patients receiving lenalidomide doses up to 50 mg, including those with reduced drug clearance due to renal impairment. In conclusion, single doses of lenalidomide up to 50 mg were not associated with prolonged QTc intervals in healthy males. The C–QTc analysis further assured that lenalidomide doses up to 50 mg are not expected to prolong QTc intervals in patients.

Lenalidomide is an oral immunomodulatory drug with proven clinical activity against a range of haematological malignancies. In combination with dexamethasone, it is currently licensed in the USA, Europe and other countries for the treatment of relapsed and/or refractory (RR) multiple myeloma (MM) 1,2. Lenalidomide in combination with dexamethasone has also been associated with good tumour response rates 4, improved progression-free survival 5,6 and/or improved overall survival 7 in patients with newly diagnosed MM; this indication is currently under clinical evaluation. In the USA and a few other countries, lenalidomide is also approved for the treatment of transfusion-dependent anaemia due to low- or intermediate-1-risk myelodysplastic syndromes (MDS) associated with a deletion 5q cytogenetic abnormality, with or without additional cytogenetic abnormalities 8. The efficacy of lenalidomide is currently being evaluated in clinical studies for other haematological cancers such as chronic lymphocytic leukaemia 9 and B-cell lymphoma 10,11.

The maximum approved therapeutic dose of lenalidomide is 25 mg daily for patients with RRMM and 10 mg daily for patients with MDS 13. The maximum clinical dose of lenalidomide for other haematological cancers is generally ≤25 mg. In terms of pharmacokinetic disposition, lenalidomide undergoes minimum metabolism, and 82% of the lenalidomide dose is eliminated unchanged via urinary excretion 14. Renal impairment reduces the elimination of lenalidomide, thereby increasing drug concentration in plasma 15. As patients with MM or MDS often have compromised renal function due to their age or comorbidities, plasma lenalidomide exposure is generally higher in these patients than in young healthy volunteers. Dose adjustment according to renal function has been recommended for patients with moderate or worse renal impairment 13.

The QT interval corrected for heart rate (HR; QTc) is a measure of ventricular repolarization on a 12-lead surface electrocardiogram (ECG). Drug-induced inhibition of human ether-a-go-go-related-gene (hERG) potassium channels has been associated with QT/QTc interval prolongation 16,17. QT/QTc prolongation can lead to an increased susceptibility to cardiac arrhythmias, in particular *torsades de pointes*, that can degenerate into ventricular fibrillation and sudden death 19–20. Therefore, regulatory agencies have emphasized the need to characterize the effect of drugs on QT/QTc intervals.

The potential risk of lenalidomide in delaying cardiac repolarization was investigated previously in non-clinical studies 21. In an *in vitro* cellular electrophysiological study, a minor (8%) inhibition of hERG current was observed at an extremely high lenalidomide concentration (204 μg/mL); this concentration is approximately 400 times greater than the mean maximum plasma concentration (*C*_max_) of lenalidomide observed in patients receiving therapeutic doses (up to 25 mg). In anaesthetized dogs, no adverse changes were observed in the cardiovascular system at single intravenous doses of lenalidomide (up to 20 mg/kg). The plasma *C*_max_ at 20 mg/kg in dogs was approximately 48 times greater than the efficacious peak plasma concentrations observed in patients. Overall, these non-clinical results suggest that the likelihood of significant QT/QTc prolongation in human beings may be very low for lenalidomide even at supratherapeutic concentrations. However, to date, the effect of lenalidomide on ventricular repolarization in human beings has not been assessed rigorously.

We conducted a thorough QT (TQT) study to evaluate the effect of lenalidomide on the QTc interval in healthy male volunteers according to the E14 guideline from the International Conference on Harmonisation (ICH) of Technical Requirements for Registration of Pharmaceuticals for Human Use 22. To confirm that the results obtained from the TQT study can be extended to targeted patient populations, we also compared the plasma lenalidomide exposure of healthy volunteers in the TQT study with that of targeted patient populations in other clinical studies and assessed the lenalidomide concentration–QTc (C–QTc) relationship.

## Methods

### TQT study

#### Study population

Sixty healthy male volunteers, aged 18–50 years, in good health as determined by medical history, physical examination and clinical laboratory tests were eligible for enrolment in this study. Body mass index (BMI) and body-weight were required to be in the ranges of 19–30 kg/m^2^ and 50–115 kg, respectively. Subjects with a history of cardiovascular disease and HR <50 or >100 beats per min. were excluded. Subjects with a QT corrected by the Bazett method (QTcB) or by the Fridericia method (QTcF) >430 ms, or with a QTcF <300 ms were also excluded.

The study was conducted in accordance with the ethical principles of Good Clinical Practice. All subjects gave written informed consent prior to enrolment. The study was approved by the institutional review boards of the participating centres and was conducted according to the Declaration of Helsinki and the ICH Guidelines for Good Clinical Practice.

#### Study design

This was a single-centre, randomized, single-dose, cross-over study conducted in the USA. The fluoroquinolone antibiotic moxifloxacin was used as a positive control in this study because of its well-characterized and consistent QTc prolongation effect 23. The study comprised four periods and four treatment sequences. Participants (*N *=* *60) were randomized into four sequences (*n *=* *15 per sequence). Each of the 60 subjects received four treatments, in a randomized order: lenalidomide 10 mg, lenalidomide 50 mg, moxifloxacin 400 mg (positive control) and placebo. All treatments were administered orally at the same time each period (approximately 08:00 on day 1 of the period) under fasting conditions together with 240 mL water. There was a 7- to 10-day washout period between treatment periods. The lenalidomide and placebo treatments were given in a double-blind manner, whereas moxifloxacin treatment was open-label to both the investigator and subjects. ECG readers were blinded to all treatments and sequences, including moxifloxacin.

#### Determination of sample size

The assumptions made in the sample size calculations for QTc assessment were as follows: (a) the within-subject standard deviation was 9.2 ms; and (b) the true difference in the time-matched and baseline-adjusted QTc between lenalidomide and placebo was 3 ms. According to these assumptions, a sample size of 49 subjects with triplicate ECGs per time-point would provide ≥80% power (for up to 10 time-points) to show that the upper limit of each two-sided 90% confidence interval (CI) for the difference in the baseline-adjusted QTc between lenalidomide and placebo would fall <10 ms. The total number of subjects was increased to 60 to account for potential drop-outs during the study.

#### Pharmacodynamic evaluation

Continuous 12-lead Holter ECG monitoring was performed for approximately 24 hr on the day before treatment (day −1) and on the treatment day (day 1) of period 1, and on day 1 of periods 2–4. On day 1 of each period, continuous ECG monitoring began 30 min. prior to drug administration and continued for ≥23 hr after administration. ECG monitoring began at a similar time on days −1 and 1 of period 1. Subjects were asked to rest in the supine position for 10 min. before and after each scheduled time-point for ECG measurement on days −1 and 1. The scheduled ECG measurements were taken at 1 hr and at 0.5 hr before drug administration, immediately before drug administration (denoted as time 0), and at 0.5, 1, 1.5, 2, 3, 4, 6, 8, 12 and 23 hr after drug administration. For a given subject, all baseline and after treatment ECGs were performed using the same ECG Holter monitor. ECGs, recorded on flash memory cards, were sent to the core ECG laboratory (Quintiles, Mumbai, India) for blinded, third-party evaluation. Triplicate ECGs, taken 2 min. apart, were extracted from the flash memory card at the scheduled time-points. ECGs were analysed for ventricular HR, PR intervals, QRS complexes, RR intervals, QT intervals and morphological changes in the ECG waveform. A single ECG reader, who was blinded to the study, was assigned to read all ECGs for a particular subject on 1 day.

The primary method used to correct QT interval for HR was the individual correction method: QTcI = QT/RR^β^, where RR is the interval between adjacent QRS complexes and β is the patient-specific correction factor. All the individual QT and RR interval pairs collected on day −1 and prior to dosing on day 1, in period 1, were used to calculate the correction factor β for QTcI. Secondary correction methods included the Fridericia method, where QTcF = QT/RR^1/3^, and the Bazett correction method, where QTcB = QT/RR^1/2^. For evaluation of QTc prolongation, triplicate QTc measures were averaged at each time-point. The baseline QTc value was defined as the average of pre-dose assessments at 1, 0.5 and 0 hr prior to drug administration on day 1 of each treatment period.

For central tendency analysis, a mixed-effect analysis of covariance (ancova) was performed on change from baseline in QTcI with fixed effects for treatment, period, sequence, scheduled time, period-specific baseline value as a covariate and the interaction of treatment by scheduled time with scheduled time as a repeated measure. The upper limit of the two-sided 90% CI was calculated for the difference in the baseline-adjusted least-squares (LS) mean QTcI between lenalidomide and placebo treatments at each time-point. If the upper limit of the two-sided 90% CI (equivalent to the upper limit of the one-sided 95% CI) at all time-points fell <10 ms (regulatory concern) 22, it was considered that the lenalidomide doses tested do not prolong the QTc interval to a clinically significant degree (i.e. negative QT study). Similar analyses were performed for QTcB and QTcF.

The effect of moxifloxacin on QTcI was evaluated at 1, 2, 3 and 4 hr after administration to assess study sensitivity. At each time-point, the difference in the baseline-adjusted LS mean QTcI between moxifloxacin and placebo treatments and the lower limit of the two-sided 97.5% CI for the difference was computed using ancova. If the lower limit of the 97.5% CI was >5 ms for at least one time-point for moxifloxacin, the study was deemed acceptable for evaluating the effect of lenalidomide on QTc.

For categorical analyses, the number and percentage of subjects with maximum QTc interval values <450, 450–480, 481–500 and >500 ms, and the number and percentage of subjects with maximum QTc interval increases from baseline to <0 or no increase, 1–30 ms, 31–60 ms and >60 ms were calculated. The results were compared between treatments.

#### Pharmacokinetic evaluation

In each treatment period, blood samples were collected from each subject before, within 5 min. of drug administration and at 0.5, 1, 1.5, 2, 3, 4, 6, 8, 12 and 23 hr after administration. After administration samples were collected within 5 min. after the nominal ECG time to avoid interference with the ECG recordings.

The plasma concentrations of lenalidomide were determined using a validated liquid chromatography-tandem mass spectrometry (LC-MS/MS) assay as described previously 24. Non-compartmental pharmacokinetic parameters were estimated from lenalidomide plasma concentration–time profiles using WinNonlin® Professional (version 5.2; Pharsight Corporation, Sunnyvale, CA, USA). The following parameters were determined: *C*_max_, time to maximum plasma concentration (*t*_max_), terminal phase plasma half-life (*t*_1/2_), area under the plasma concentration–time curve from time 0 to infinity (AUC_∞_), apparent total body clearance after oral dosing (CL/F) and apparent volume of distribution in terminal phase (V_z_/F).

#### Lenalidomide concentration–QTc relationship

The relationship between individual changes from placebo in the baseline-adjusted QTcI values (ΔΔQTcI) and the time-matched lenalidomide concentrations in plasma was examined graphically. The relationship was further tested via a linear mixed-effect model, with the lenalidomide concentration as the independent variable and ΔΔQTcI as the dependent variable. Subjects were included as a random effect for the slope in the linear mixed-effect model.

#### Safety evaluation

The safety and tolerability of single doses of lenalidomide were assessed throughout the study by monitoring adverse events (AEs), vital signs, clinical laboratory values (haematology, blood chemistry, urinalysis) and standard 12-lead ECGs.

#### Pharmacokinetic studies in patients

Lenalidomide pharmacokinetics were evaluated in patients with MM (5, 10, 25 and 50 mg doses) and in patients with MDS (10 mg dose), and the study designs have been reported separately 25–26. Briefly, blood samples were collected before dose administration and at 0.25, 0.5, 0.75, 1, 1.5, 2, 2.5, 3, 4, 6, 8, 10, 12, 18 and 24 hr after the first dose. The lenalidomide concentration in plasma was determined using validated LC-MS/MS methods as described previously 15–27. Non-compartmental pharmacokinetic parameters were estimated as described for the TQT study.

## Results

### TQT study

#### Subject demographics

A total of 60 subjects were enrolled (*n *=* *15 per sequence), with 52 subjects completing ECG assessments in all four treatment periods. The mean age of the subjects was 28 years (range 18–49 years), the mean weight was 80.1 kg (range 58.8–103.8 kg), and the mean BMI was 25.1 kg/m^2^ (range 19.1–29.9 kg/m^2^). Most (63.3%) of the subjects were white; 30.0% were black. The 52 subjects who completed the ECG assessments in all four treatment periods were included in the pharmacodynamic analysis.

#### Pharmacodynamics

##### Correction of QT intervals

Three methods were used to correct the QT interval for HR. The primary method, individual correction, adequately corrected QT intervals over the observed HR range as demonstrated by the lack of any trend between QTcI and RR intervals. The mean individual correction factor for the QT interval was determined to be 0.29, lower than both the Fridericia correction factor (0.33) and the Bazett correction factor (0.5). The mean baseline value in period 1 was 399 ms for QTcI, 398 ms for QTcF and 395 ms for QTcB.

##### Central tendency analysis

**Figure 1:**
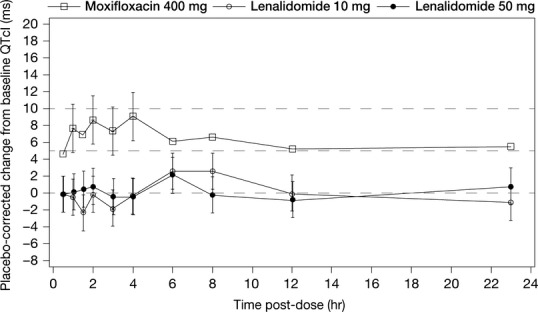
Least-squares (LS) mean and 90% two-sided confidence intervals (CIs) for time-matched baseline-adjusted individual corrected QT (QTcI) change from placebo after single oral doses of lenalidomide in healthy volunteers. Data from the positive control moxifloxacin are included for comparison and are shown as LS mean and 97.5% two-sided CIs.

**Figure 2:**
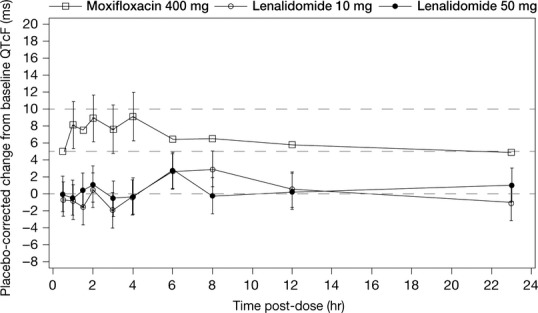
Least-squares (LS) mean and 90% two-sided confidence intervals (CIs) for Fridericia-adjusted QTc (QTcF) change from placebo after single oral doses of lenalidomide in healthy volunteers. Data from the positive control moxifloxacin are included for comparison and are shown as LS mean and 97.5% two-sided CIs.

**Figure 3:**
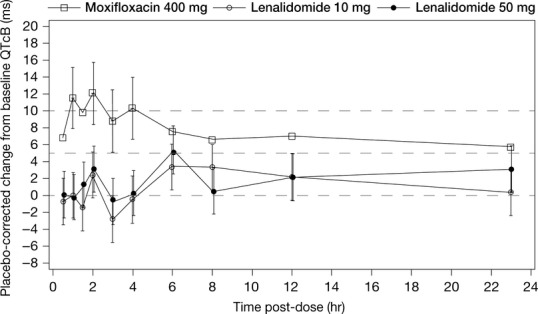
Least-squares (LS) mean and 90% two-sided confidence intervals (CIs) for Bazett-adjusted QTc (QTcB) change from placebo after single oral doses of lenalidomide in healthy volunteers. Data from the positive control moxifloxacin are included for comparison and are shown as LS mean and 97.5% two-sided CIs.

During the moxifloxacin treatment period, the change from placebo in the baseline-adjusted LS mean QTcI values was >7 ms at all time-points between 1 and 4 hr after administration. The lower limit of the associated two-sided 97.5% CI was >5 ms at 2 and 4 hr after dosing (fig. 1). Similar changes were observed for QTcF and QTcB values (fig. 1). Assay sensitivity was therefore considered to be established for the evaluation of the effect of lenalidomide on QT intervals corrected by either primary or secondary methods. For single doses of lenalidomide (10 and 50 mg), the change from placebo in the baseline-adjusted LS mean QTcI values was <3 ms, with the upper limit of the two-sided 90% CI remaining <10 ms at all time-points. The changes from placebo in the baseline-adjusted LS mean QTcF or QTcB values for lenalidomide 10 and 50 mg were also <3 ms, and the upper limit of the two-sided 90% CI was <10 ms at all time-points measured (figs 2 and 3).

##### Categorical and waveform analyses

No subject had a maximum QTcI, QTcF or QTcB value >450 ms after receiving lenalidomide or placebo. One subject had a QTcI value of 450–480 ms after receiving moxifloxacin.

No subject showed an increase from baseline in the QTcI or QTcF value >30 ms after receiving lenalidomide, placebo or moxifloxacin. An increase from baseline in the QTcB value 30–60 ms was recorded after moxifloxacin (*n *=* *6 subjects), placebo (*n *=* *2 subjects), lenalidomide 10 mg (*n *=* *2 subjects) and lenalidomide 50 mg (*n *=* *3 subjects). An increase from baseline of the QTcB value >60 ms was not reported for any subjects.

The only new-onset cardiac abnormality observed during the study was abnormal T wave, which was observed in two subjects in the placebo period and in three subjects each in the lenalidomide and moxifloxacin periods.

#### Pharmacokinetics

**Figure 4:**
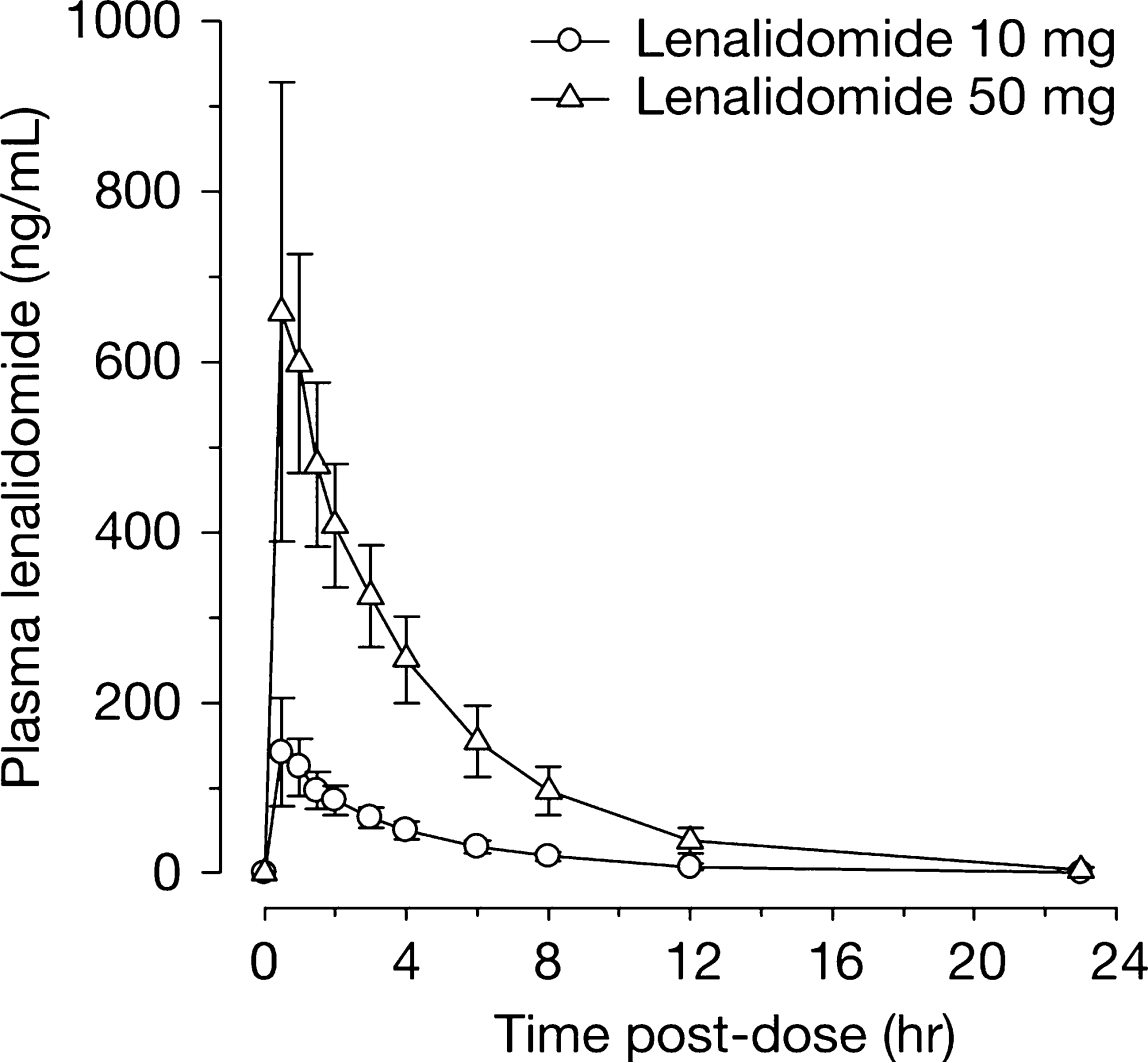
Mean (± standard deviation) plasma lenalidomide concentrations over time in healthy volunteers.

Mean plasma lenalidomide concentration profiles are presented in fig. 4. In this study, *C*_max_ was observed at a median time of 0.53 hr after dosing and the mean half-life (*t*_1/2_) was approximately 3 hr for both doses. Consistent with the linear pharmacokinetics of lenalidomide 27, plasma exposure to lenalidomide (*C*_max_ and AUC_∞_) was proportional to the dose in these healthy volunteers.

#### Relationship between QTc and lenalidomide concentrations

**Figure 5:**
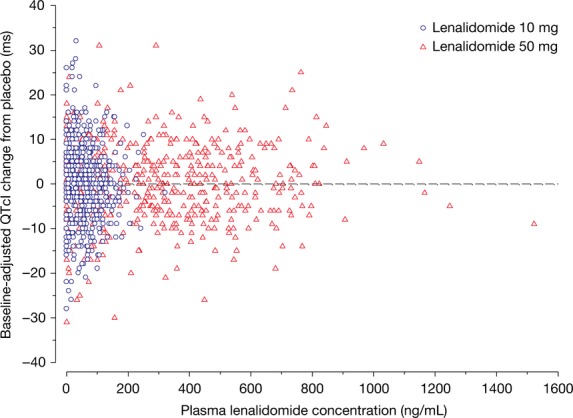
Plasma lenalidomide concentrations *versus* the time-matched baseline-adjusted individual corrected QT (QTcI) change from placebo in healthy volunteers.

As shown in fig. 5, individual ΔΔQTcI values were distributed randomly around zero across lenalidomide concentrations up to 1522 ng/mL. Further analyses using a linear mixed-effect model yielded a very small slope of approximately 0.0005 ms ng/mL that was statistically insignificant from zero (90% CI: −0.00167 to 0.00273 ms ng/mL), confirming no significant relationship between ΔΔQTcI values and lenalidomide concentrations.

#### Safety

No deaths or serious AEs occurred during this study. Three subjects reported AEs associated with the cardiovascular system (intermittent mild palpitations, Wolff–Parkinson–White syndrome and syncope, respectively), none of which was considered to be related to the treatment. The most common treatment-related AE was headache (3.3%) reported by one subject treated with moxifloxacin and one subject treated with lenalidomide 50 mg. Other treatment-related AEs included nausea, drug hypersensitivity, elevated alanine transferase (ALT) levels and sleep disorders. One subject left the study due to a moderate elevation of ALT levels after the placebo treatment in period 2. The highest number of AEs was reported during the moxifloxacin treatment period (12/56 subjects, 21.4%) and the lowest number during placebo treatment (4/57 subjects, 7%). A similar proportion of subject reported ≥1 AE during lenalidomide 10 mg (9/56 subjects, 16.1%) and 50 mg (8/54 subjects, 14.8%) treatments. All AEs were mild or moderate in severity.

### Pharmacokinetic studies in patients

#### Patient demographics

The mean age was 58 years (range 40–73 years) for patients with MM and 69 years (range 47–85 years) for patients with MDS; approximately 33% of patients with MM and MDS were aged ≥65 years. Almost all (98%) of the patients with MM and MDS were white, with approximately 35% female. Mild-to-moderate renal impairment was found in approximately 42% of patients with MM and 58% of patients with MDS.

#### Pharmacokinetics

The plasma exposure parameters of lenalidomide for patients with MM and MDS after a single dose are presented in table 1. Both *C*_max_ and AUC were approximately proportional to doses in patients with MM (5–50 mg). At the same 10 mg dose, the exposure level was comparable between patients with MM and MDS.

**Table 1:** Plasma lenalidomide exposure parameters in healthy volunteers and in patients with MM or MDS

*C*_max_ (ng/mL)					AUC_∞_ (hr ng/mL)	
Disease	Dose (mg)	Patients (n)	Geometric mean (CV%)	Range	Geometric mean (CV%)	Range
Healthy	10	55	155 (30.1)	76–321	536 (18.7)	369–974
	50	54	698 (31.1)	301–1522	2674 (19.8)	1853–5013
MM	5	6	99 (47.4)	58–213	535 (58.5)	358–1484
	10	10	208 (41.9)	100–419	989 (57.7)	620–3354
	25	6	555 (17.5)	446–740	2253 (24.8)	1593–2956
	50	20	994 (33.3)	603–2413	4663 (38.3)	2343–11,647
MDS	10	12	179 (32.6)	110–286	933 (32.6)	574–1733

AUC_∞_, area under the curve from time zero to infinity; *C*_max_, maximum plasma concentration; CV, coefficient of variance; MDS, myelodysplastic syndromes; MM, multiple myeloma.

These exposure parameters were compared with those observed in healthy volunteers from the TQT study (table 1). At the same lenalidomide dose level (10 or 50 mg), patients had a higher exposure than young healthy volunteers, a finding that is consistent with their age- and disease-related compromised renal function. Nevertheless, individual values of lenalidomide *C*_max_ at the approved therapeutic doses (up to 25 mg) ranged from 58 to 740 ng/mL. This *C*_max_ range was within that evaluated in the TQT study (76–1522 ng/mL). In addition, all but two patients with MM treated with 50 mg lenalidomide had a *C*_max_ value below the maximum *C*_max_ value (1522 ng/mL) observed in the TQT study; these two had *C*_max_ values of 1749 and 2413 ng/mL, respectively.

The geometric mean of the *C*_max_ at the 50 mg dose was 994 ng/mL, which, utilizing the C–QTc model obtained from the TQT study, would be associated with approximately a 0.52 ms increase in QTcI (corresponding to a slope of 0.0005 ms ng/mL). If the upper limit of the two-sided 90% CI for the C–QTc relationship was used, the increase in QTc for the 50 mg dose would be 2.71 ms (corresponding to a slope of 0.00273 ms ng/mL), which was far below the threshold of the regulatory concern (10 ms).

## Discussion

The objective of the TQT study was to provide a rigorous assessment of the potential for lenalidomide to prolong the QTc interval in human beings across the therapeutic and supratherapeutic concentration ranges. The primary hypothesis compared the change in QTc interval from pre-dose baseline values between lenalidomide and placebo treatments. In this study, there was no detectable increase in QTc at any time-point studied after lenalidomide administration.

The TQT study was carried out in healthy volunteers to circumvent many of the obstacles to QTc evaluation encountered in patients with cancer. Patients with MM or MDS are older (often elderly), have multiple comorbidities (e.g. hypercholesterolaemia, hypertension, renal impairment, anaemia) and receive concurrent medications including those associated with QTc prolongation. These patients may also be at increased risk of severe cardiac arrhythmias due to treatment-related factors such as electrolyte imbalances (hypokalaemia, hypocalcaemia), poor nutritional status and the effects of supportive medications (antiemetics, antihistamines) on QTc 28. Performing a QT interval study in healthy volunteers allows the collection of robust ECG and pharmacokinetic data, minimizes confounding factors and uses a positive control, all of which facilitate data interpretation.

Single doses were chosen in this study because lenalidomide and its major metabolites (<5% of the parent compounds in plasma) do not accumulate over time in plasma 27–29. The lenalidomide dose levels of 10 and 50 mg were chosen to cover a broad concentration range similar to that observed in clinical trials with patients. A higher dose, causing a plasma concentration in substantial excess of that observed in the targeted patient population, was not used in this study due to non-clinical toxicity profile considerations.

The sensitivity of the study in detecting a modest increase in QTc interval was established with the positive control moxifloxacin at a dose of 400 mg. The time-matched changes from placebo in the baseline-adjusted LS mean QTcI interval were between 7 and 10 ms 1–4 hr after moxifloxacin administration, with the lower limit of the two-sided 97.5% CI >5 ms at 2 and 4 hr. The timing and magnitude of QTc prolongation after the administration of moxifloxacin was consistent with the response typically reported for this positive control 23. Thus, the study has demonstrated the ability to detect a small QTc prolongation.

For lenalidomide doses of 10 and 50 mg, all time-matched changes from placebo in the baseline-adjusted LS mean QTcI interval were <3 ms, with the upper limit of the two-sided 90% CI staying substantially <10 ms. The central analyses also showed negative results using QTcF and QTcB data. In addition, there were no extreme QTc values (QTc >450 ms or QTc change from baseline >60 ms) after lenalidomide treatment. The ICH E14 definition of a negative TQT study 22 was thereby met.

Regulatory guidance requires examination of QT/QTc prolongation at supratherapeutic doses to account for increased systemic drug concentrations, which may be encountered in clinical practice. Lenalidomide at a dose of 50 mg is twice the approved maximum therapeutic dose for MM (25 mg) and five times that for MDS (10 mg). The mean *C*_max_ (698 ng/mL) achieved at the 50 mg dose in the TQT study was approximately 26% more than that observed in patients with MM (555 ng/mL at 25 mg), and 390% greater than the *C*_max_ reported in patients with MDS (179 ng/mL at 10 mg) (table 1). In the TQT study, individual lenalidomide *C*_max_ in plasma ranged from 76 to 1522 ng/mL, and no statistically significant or clinically important relationship was observed between drug concentration and QTcI change. The *C*_max_ range observed in the TQT study was close to that observed in patients receiving lenalidomide doses up to 50 mg (58 to 2413 ng/mL, with only two patients having a *C*_max_ >1500 ng/mL). Further, utilizing the C–QTc model obtained from the TQT study, the upper limit of the two-sided 90% CI for the changes in QTcI at the geometric mean *C*_max_ (994 ng/mL) was predicted to be far below the 10 ms threshold in patients with MM receiving the supratherapeutic dose (50 mg).

In the absence of dose adjustment, the most likely condition associated with elevated lenalidomide exposure in plasma is renal impairment. Chen *et al*. 15 reported individual *C*_max_ values ranging from 376 to 1047 ng/mL (25 mg dose) in patients with various degrees of renal impairment, a range that is within the *C*_max_ range evaluated in the current TQT study. Thus, even at the maximum therapeutic dose (25 mg), lenalidomide concentrations in patients with renal impairment are not expected to exceed the highest level observed in the TQT study.

In patients with MM or MDS with comorbidities, lenalidomide may be coadministered with antiarrhythmics, antibiotics, antiemetics, antihistamines and other chemotherapies, some of which are known to be associated with QT prolongation 30. However, pharmacokinetic interactions are not anticipated between lenalidomide, and most of these comedications because lenalidomide is neither a substrate nor an inhibitor of the major cytochrome P450 enzymes that are responsible for elimination of many QT-prolonging drugs 31. In addition, lenalidomide has little effect on the hERG current *in vitro* at concentrations far above the clinical concentration range 21; interaction with other drugs at the hERG potassium channels is also unlikely to occur. Thus, when lenalidomide is coadministered with a QT-prolonging drug, the likelihood of an increased risk in QT prolongation is considered to be low.

MM and MDS are diseases occurring at advanced age, and most patients with MDS associated with a deletion 5q cytogenetic abnormality are female. These two populations (female sex and advanced age) are more susceptible to drug-induced long QT syndrome and *torsades de pointes* 30. Although the current TQT study excluded females and the elderly due to safety considerations, in pivotal lenalidomide trials, the median age of patients with MM was approximately 63 years with 40% of them are female. The median age of patients with MDS was approximately 70 years with over two-thirds female [data on file]. Data from these patients treated with lenalidomide did not reveal any concerns with regard to QTc prolongation 1–8. Also, as mentioned earlier, the *C*_max_ range observed in the TQT study was close to that observed in the pharmacokinetic studies of patients (35% female and approximately 33% elderly) receiving lenalidomide doses up to 50 mg (the supratherapeutic dose). Given the negative QTc findings over this wide *C*_max_ range, it is unlikely that lenalidomide would cause clinically relevant QTc prolongation in female and elderly patients.

When taken together, the negative QTc findings from the central and categorical analyses, the flat C–QTcI relationship and the known drug concentration ranges in patients, all support that lenalidomide up to 50 mg is unlikely to cause QTc prolongation in the targeted patient population.

In conclusion, single doses of lenalidomide 10 and 50 mg were not associated with prolongation of the QT interval in healthy male patients. The C–QTc analysis further confirmed that lenalidomide doses up to 50 mg are not expected to prolong QTc intervals in patients.
